# Calcium and Ca^2+^-Binding Proteins Regulate Microtubule and Cytoskeletal Dynamics During Mammalian Corticogenesis

**DOI:** 10.3390/brainsci16050499

**Published:** 2026-05-01

**Authors:** Diana Sarahi De la Merced-García, Rocío Valle-Bautista, Rebeca Hernández-García, Néstor Fabián Díaz, Anayansi Molina-Hernández

**Affiliations:** 1 Departamento de Fisiología y Desarrollo Celular, Instituto Nacional de Perinatología Isidro Espinosa de los Reyes, Montes Urales 800, Lomas Virreyes, Miguel Hidalgo, Mexico City 11000, Mexico; dlmgarciads@gmail.com (D.S.D.l.M.-G.); rociovb26@gmail.com (R.V.-B.); reb98eca@gmail.com (R.H.-G.); nfabian00@gmail.com (N.F.D.); 2 Doctorado en Ciencias Biomédicas UNAM, Unidad de Posgrado Edificio B Primer Piso Ciudad Universitaria, Mexico City 04510, Mexico; 3 Posgrado en Ciencias Biológicas, Unidad de Posgrado, Edificio A, 1° Piso, Circuito de Posgrados, Ciudad Universitaria, Coyoacán, Mexico City 04510, Mexico

**Keywords:** calcium signaling, corticogenesis, microtubules, neural stem cell proliferation, neuronal migration, neurite growth

## Abstract

Intracellular calcium (Ca^2+^) signaling is a central regulator of corticogenesis, governing haveneural stem cell behavior, fate transitions, neuronal migration, and circuit assembly. Beyond its canonical role as a second messenger, Ca^2+^ shapes cytoskeletal organization by modulating microtubule dynamics essential for mitotic spindle function, radial glial scaffold, nucleokinesis, and neurite extension. This review synthesizes evidence from in vivo, ex vivo, and in vitro studies to delineate Ca^2+^-dependent pathways and Ca^2+^-binding proteins that couple, within restricted Ca^2+^ microdomains in space and time, to microtubule regulation during mammalian cortical development. We highlight mechanistic nodes involving calmodulin, Ca^2+^/calmodulin-dependent kinases (CaMKs), S100 proteins, cadherins/protocadherins, centrins (CENs), and Ca^2+^ sensors such as STIM1 and calneurons, which collectively coordinate spindle orientation, progenitor division modes, radial migration, and neurite outgrowth. Finally, we discuss how perturbations in Ca^2+^-controlled cytoskeletal programs may contribute to abnormal cortical cytoarchitecture and neurodevelopmental disease. By integrating Ca^2+^ microdomain transients with microtubule control modules, this review provides a unified framework for understanding how Ca^2+^ orchestrates key cellular events during mammalian corticogenesis and propose that Ca^2+^ oscillatory codes are translated into direct or indirect microtubule/cytoskeletal remodeling transitions that determine neural stem cell fate, migration, and maturation, to accurately establish cortical architecture and function.

## 1. Introduction

Corticogenesis is a tightly regulated process that encompasses proliferation and differentiation of neural stem cells (NSCs), neuronal migration, and the establishment of the laminated architecture of the cerebral cortex. During early neural tube development, neuroepithelial cells (NECs) undergo symmetric divisions to expand the NSC pool. As development progresses, NECs acquire a radial glial cell (RGC) identity, retaining self-renewal and multilineage differentiation capacity, and provide a scaffold to guide neuroblast migration towards the developing cortical plate [1].

Intracellular calcium (Ca^2+^) is a key regulator of NSC proliferation and fate decision during cortical development. In early corticogenesis, Ca^2+^ signaling modulates RGC polarity, proliferation, differentiation, and neuroblast migration. Ca^2+^ signaling displays spatiotemporal oscillatory patterns that trigger distinct cellular responses, including cell cycle progression, differentiation, neuronal migration, and maturation [2,3].

**Figure 1:**
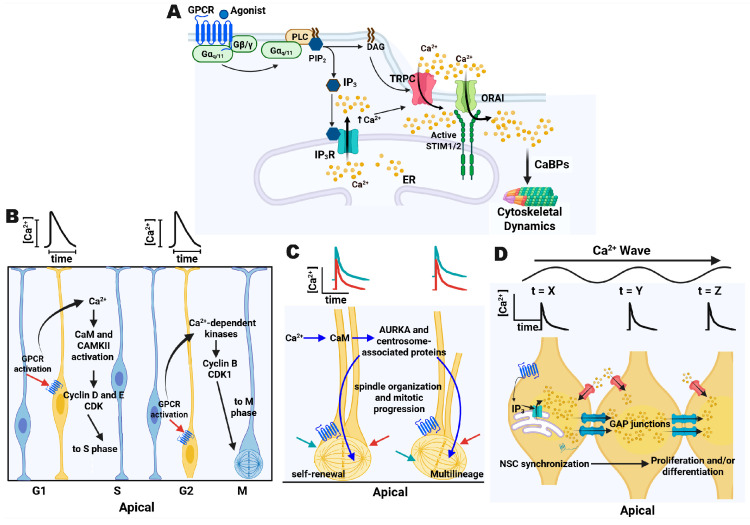
G-protein-coupled-receptor-dependent Ca^2+^ entry and Ca^2+^ transient patterns in neural stem cells. (**A**) Downstream signaling after agonist binding to a Gα_q/11_-coupled G-protein-coupled receptor (GPCR). GPCR activation stimulates phospholipase C (PLC), generating IP3 and DAG, triggering Ca^2+^ release from the endoplasmic reticulum (ER) via IP3 receptors, which further promotes Ca^2+^ influx through transient receptor potential canonical (TRPC) and Ca^2+^ release-activated Ca^2+^ (CRAC; ORAI) channels. Elevated Ca^2+^ engages Ca^2+^-binding proteins (CaBPs) that regulate cytoskeletal dynamics. (**B**–**D**) Representative patterns of spontaneous Ca^2+^ transients observed in cortical neuroepithelium/radial glia and their related pathways: (**B**) slow, sporadic, unsynchronized oscillations involving Ca^2+^/calmodulin (CaM), Ca^2+^/calmodulin-dependent kinases (CaMKs), cyclin-dependent kinase (CDK) for growing phase 1 (G1) to synthesis (S) and for growing phase 2 (G2) to mitosis/cytokinesis (M) transitions; (**C**) synchronous Ca^2+^ increase between dividing sister cells involving CaM and Aurora kinase A (AURKA); and (**D**) multicellular Ca^2+^ waves propagated through gap junctions through time (t). For (**B**–**D**), the upper traces depict representative Ca^2+^ transients, and cell color indicates relative intracellular Ca^2+^ levels (yellow, elevated; blue, low). Created in https://BioRender.com/aiiw8bj (accessed on 18 April 2026).

In non-excitable cells, such as RGCs, a prominent mechanism driving cytosolic Ca^2+^ elevation is activation of G_αq11_ by G-protein-coupled receptors (GPCRs). This pathway involves activation of phospholipase C (PLC), which hydrolyzes phosphatidylinositol 4,5-bisphosphate into inositol 1,4,5-trisphosphate (IP_3_) and diacylglycerol (DAG). IP_3_ binds to its receptors (IP_3_Rs) on the endoplasmic reticulum (ER), triggering Ca^2+^ release from the ER lumen into the cytosol, promoting additional Ca^2+^ entry and the activation of downstream intracellular pathways (Figure 1A) [4,5].

Studies in rat embryonic cortical neuroepithelium have described three patterns of spontaneous Ca^2+^ activity in RGCs that depend on ER Ca^2+^ release mediated by IP_3_Rs, extracellular Ca^2+^ influx via transient receptor potential channels (TRPC), and Ca^2+^ influx through Ca^2+^-release-activated calcium (CRAC) channels. The first pattern comprises slow (~40 s), sporadic, and unsynchronized oscillations at the single-cell level, which have been linked to G_1_–S or G_2_–M transitions (Figure 1B) [6,7]. Experimental evidence indicates that slow Ca^2+^ oscillations are associated with cell-cycle progression and are likely decoded by Ca^2+^-dependent regulators, such as calmodulin (CaM) and Ca^2+^/calmodulin-dependent kinases (CaMKs), which can modulate cyclin-dependent programs and key cell-cycle transitions [8]. A second pattern consists of synchronous Ca^2+^ increases between dividing sister cells, suggesting a potential role in mitotic spindle orientation or cytokinesis (Figure 1C) [6]. In this context, coordinated Ca^2+^ transients are compatible with activation of spindle-regulatory machinery, including Aurora-A kinase (AURKA) and centrosome-associated components, which are influenced by Ca^2+^/CaM signaling and are required for correct spindle organization and mitotic progression [9]. A third pattern involves multicellular Ca^2+^ events mediated by intracellular wave propagation through gap junctions (Figure 1D). These multicellular waves have been proposed to promote NSC synchronization within the ventricular zone (VZ), likely via intercellular coupling mechanisms that coordinate proliferation and early neurogenesis [7,10,11]. Although the precise decoding modules are not fully defined in these paradigms, the available data support a framework in which distinct spatiotemporal Ca^2+^ signatures engage specific molecular pathways controlling cell-cycle progression, spindle behavior, and progenitor coordination during corticogenesis.

These phenomena reflect a structured spatial organization in which Ca^2+^ operates within signaling microdomains composed of effectors, regulators, and substrates, assembled by scaffolding/anchoring proteins. Ca^2+^ binds directly to a broad range of proteins, including those that regulate Ca^2+^ dynamics (pumps, channels, and buffer proteins) and those that mediate downstream processes such as enzymatic activation, cytoskeletal remodeling, and transcriptional control. The specificity of Ca^2+^ signaling during corticogenesis is therefore determined by signal duration, amplitude, oscillation frequency, spatial localization, and timing [12].

This review focuses on the mechanisms by which Ca^2+^ signaling regulates microtubule dynamics during mammalian corticogenesis, emphasizing its roles in NSC division, neuronal migration, and neurite outgrowth. We further examine how distinct Ca^2+^ oscillation features (frequency, amplitude, and spatial confinement with microdomains) may be decoded by Ca^2+^-binding proteins (CaBPs) to modulate microtubule stability and remodeling across successive stages of cortical development, from progenitor proliferation and neuronal differentiation to migration and neurite maturation.

It is important to note that other ions, including Na^+^, K^+^, and Cl^−^, as well as divalent cations such as Mg^2+^ and Zn^2+^, also contribute to neurodevelopmental processes. Monovalent ions regulate membrane excitability, osmotic balance, and aspects of stem cell physiology [13]. Mg^2+^ has been implicated in oligodendrocyte differentiation, in part through modulation of NMDA receptor function [14]. Zn^2+^ influences proliferation, survival, migration, and differentiation by regulating enzyme activity, transcription factor binding, and chromatin/DNA stability [15]. However, in contrast to these ions, Ca^2+^ functions as a uniquely versatile second messenger that encodes information through spatiotemporal dynamics, coupling extracellular and intracellular cues to signaling pathways that control cytoskeletal organization, gene expression, and cell-fate decisions in the developing cerebral cortex.

## 2. Ca^2+^-Binding Protein

During corticogenesis, the cytoskeleton is fundamental to proliferation, differentiation, migration, and neuronal maturation. Within the cytoskeleton, microtubules (MTs) are highly dynamic polymers whose regulated assembly and disassembly support these processes. The effect of Ca^2+^ on cytoskeleton polymerization/depolymerization depends on local concentration, the repertoire of CaBPs expressed, and the organization of subcellular Ca^2+^ microdomains [16,17,18,19].

CaBPs implicated in cytoskeletal and MT organization during neurodevelopment include CaM, Ca^2+^/CaM-dependent kinases (CaMKs), S100 proteins, parvalbumin, CEN, calbindin-D28k, gelsolin, neurocalcin, calcineurin (CaN), and annexins [19]. Key examples are summarized below.

### 2.1. CaM and CaMKs

CaM is the most abundant and best-characterized Ca^2+^ sensor in eukaryotic cells. It regulates processes such as growth, differentiation, proliferation, survival, and motility by modulating numerous enzymes, ion channels, and cytosolic-, nuclear-, and membrane-associated proteins [20].

In mammals, CaM is encoded by three genes (*CAM1*, *CAM2*, and *CAM3*) located on different chromosomes, which encode the same 149-amino-acid protein [20,21]. Structurally, CaM adopts a dumbbell-shaped conformation with N- and C-terminal globular domains connected by a central α-helix. Each globular domain contains two EF-hand motifs, enabling CaM to bind four Ca^2+^ ions [22].

CaM constitutes at least 0.1% of total cellular proteins (10^−6^–10^−5^ M), and its abundance is often higher in dividing and differentiating cells such as NSCs. CaM function is regulated through three main mechanisms: (1) its subcellular localization (cytoplasmic vs. nuclear) that enables compartment-specific effects; (2) its interaction with >300 target proteins, which is facilitated by Ca^2+^-dependent conformational changes that expose a hydrophobic binding surface; and (3) changes in affinity concentrations (from 5 × 10^−7^ to 5 × 10^−6^ M), with the C-terminal site exhibiting 3–5 times higher affinity than the N-terminal site [23].

Among the most prominent CaM effectors are CaMK family members, which exert major control over cytoskeletal dynamics. The family includes CaMKI, CaMKII, CaMKIII, and CaMKIV. Where CaMKIII primarily phosphorylates eEF2, other isoforms phosphorylate diverse substrates involved in cell morphology and plasticity [24,25]. The CaMKIIβ subunit directly interacts with filamentous actin (F-actin). Upon Ca^2+^ influx, CaMKII activation promotes a transient actin reorganization followed by return to an autoinhibited state, which can stabilize newly established cytoskeletal architecture. Although CaMKII is classically associated with actin, it also modulates microtubule-associated proteins, supporting coordinated regulation of both cytoskeletal systems [26,27].

### 2.2. S100 Proteins

The S100 family includes CaBPs, with two EF-hand motifs connected by a hinge region, enabling conformational transduction upon ion binding. The family includes 25 functional members, with intracellular and extracellular functions. S100 proteins are vertebrate-specific and display cell-type- and developmental-stage-specific expression, often at high abundance. Among its target proteins are enzymes, cytoskeletal components, receptors, transcription factors, and nucleic acids [28,29,30].

During corticogenesis, S100 proteins contribute to cell proliferation, differentiation, cell morphology, and migration. Among CNS-expressed family members, S100B and S100A4 are prominent during development. S100B has been implicated in regulating protein phosphorylation, energy metabolism, and cytoskeletal dynamics, thereby influencing cell morphology and behavior [31,32]. During corticogenesis, it is highly expressed in proliferative RGCs during neurogenesis and early differentiation, decreases during neuronal precursor differentiation, and later re-emerges in glial precursors, reaching the highest levels in astrocytes [33,34]. It has been reported that S100B can regulate microtubule dynamics both directly and indirectly: directly by promoting Ca^2+^-dependent microtubule disassembly, and indirectly by modulating protein-kinase-II-dependent phosphorylation of tau, a key microtubule-associated protein [35,36]. In human NSCs, S100B has been proposed to promote tau hyperphosphorylation via JNK signaling, thereby activating nuclear AP-1/c-Jun-dependent transcription. In this context, JNK activation has also been linked to increased expression of Dickopff-1, which promotes glycogen synthase kinase 3β activity and β-catenin degradation, ultimately reducing tau affinity for MTs and contributing to cytoskeletal destabilization [37]. However, direct experimental evidence connecting S100B to microtubule regulation, specifically in NSC division, polarity, or ventricular zone dynamics, remains limited. Future studies should therefore test whether developmental shifts in S100B expression (high-to-low) interact with Ca^2+^ signaling dynamics to differentially tune MT stability during proliferative and migratory stages, when rapid remodeling is required, versus more differentiated states, where neuronal morphology is comparatively stabilized.

S100A4, also known as metastasin or fibroblast-specific protein 1, is expressed in multiple CNS cell types during development, including neurons and RGCs [38]. In the adult CNS, S100A4 expression is generally low and has been associated with migratory behavior and cytoskeletal reorganization through interactions with actin filaments, tropomodulins, tropomyosins, liprin β1, and ezrin. Many of these interactions are Ca^2+^-dependent, although Ca^2+^-independent interactions have also been reported [39]. Functional information on S100A4 in corticogenesis remains scarce. Similar to S100B, S100A4 exhibits a prenatal spatiotemporal expression pattern, reported prominently in the human hippocampus and temporal cortex, and declines with age [40]. Notably, other S100 family members, such as S100A5 and S100A13, are expressed at higher levels in the developing brain [40], but their roles during corticogenesis remain largely unknown.

Exogenous S100A4 administration to neuron–astrocyte co-cultures has been reported to enhance neurite outgrowth and promote survival following apoptotic challenge in hippocampal, dopaminergic, and cerebellar neurons, in a RAGE-independent manner [41,42]. These findings suggest potential involvement of S100A4 in neuritogenesis during development; however, the contribution of extracellular Ca^2+^, the mechanisms governing S100A4 release from specific cell types, and whether S100A4 directly regulates cytoskeletal dynamics or fate decisions during corticogenesis remain unresolved. Collectively, these gaps highlight clear priorities for future mechanistic studies.

### 2.3. Cadherins

Cadherins are Ca^2+^-dependent cell–cell adhesion molecules with five repeated extracellular cadherin domains (EC), a single transmembrane domain, and a C-terminal cytoplasmic domain [43]. They are broadly classified into classical cadherins, clustered protocadherins, and non-clustered protocadherins [44]. Protocadherin 19 (PCDH19) is a protocadherin superfamily member characterized by a conserved 17-amino acid cytoplasmic motif. In mammals, *PCDH19* is X-linked and encodes a transmembrane protein with four extracellular cadherin repeats (EC1–EC4), a transmembrane segment, two cytoplasmic domains, and at least one nuclear localization signal [45,46].

PCDH19 EC1–EC4 domains adopt the canonical cadherin fold, including a “Greek key”-like motif composed of β-sheets forming a sandwich-like structure. The EC1 domain includes a disulfide bridge characteristic of clustered protocadherins and an α-helix in the B–C loop. Linker regions between EC1–EC2, EC2–EC3, and EC3–EC4 contain canonical Ca^2+^-binding sites critical for structural stability. This architecture supports Ca^2+^-dependent adhesion consistent with the roles of neuronal column organization reported in the zebrafish optic tectum, suggesting it may also participate in columnar neuron organization in cortical development [47].

*Pcdh19* is expressed in both the developing and adult brain [46,48,49]. During corticogenesis, its expression initiates in NECs and expands across the neuroepithelium at the onset of neurogenesis. At the neurogenic peak, *Pcdh19* is enriched in RGCs and intermediate progenitors (IPs), two proliferative cell populations: the former is multipotent, and the latter is largely neuron-restricted [50]. Intracellular Ca^2+^ transients enable Pcdh19 to regulate cell–cell contacts within the neuroepithelium. Proteomic studies further indicate that Pcdh19 interacts with proteins implicated in microtubule architecture, cell polarity, chromatid segregation, and the G2/M transition, supporting a model in which Pcdh19 coordinates, in a Ca^2+^-dependent manner, actin and microtubule dynamics to ensure proper NSCs division, migration, and differentiation during corticogenesis [51].

Cadherin-mediated adhesion interfaces with Rho family GTPases, including RhoA, RAC1, and CDC42, which coordinate actin and microtubule remodeling and are essential for establishing apico–basal polarity [52]. Importantly, cadherin-based junctions can also recruit polarity and spindle-orientation machinery, such as the LGN/NuMA complex, thereby coupling extracellular adhesion cues to centrosome positioning and mitotic spindle alignment and influencing the balance between symmetric proliferative and asymmetric neurogenic division [53,54]. Although direct evidence linking Ca^2+^-dependent adhesion mediated specifically by PCDH19 to spindle orientation in RGCs remains limited, these observations support a framework in which Ca^2+^-regulated cell–cell adhesion is transduced into intracellular polarity signals that shape cytoskeletal organization and division orientation during corticogenesis.

### 2.4. Centrins

CENs (caltractins) are small acidic phosphoproteins (~170 amino acids). Mammals encode four isoforms (CEN1–CEN4). CENs are CaM-like with N- and C-terminal globular domains, connected by a flexible hinge that yields a dumbbell-like conformation. They contain four EF-hand motifs, from which typically EF-3 and EF-4 are active. Ca^2+^ binding induces conformational rearrangements that expose hydrophobic surfaces, facilitating dimerization, oligomerization, and interactions with signaling and cytoskeletal proteins. The flexible N-terminal region (notably in CEN2) contributes to Ca^2+^-dependent self-assembly [55].

In various cell types, including NSCs, CENs associate with centrioles, key components of the centrosome. CEN1 is primarily expressed in male germ cells, whereas CEN2–CEN4 are broadly expressed in somatic cells. CEN2 localizes to centrioles throughout the cell cycle, whereas CEN3 is enriched in the pericentriolar material. During mitosis, CEN associates with the centrosome, a key structure that organizes the cytoskeleton, to serve as a nucleation and anchoring site for MTs. The centrosome comprises a centriole pair and pericentriolar material containing γ-tubulin (γ-Tub), pericentrin, and ninein [56,57,58,59,60].

## 3. Ca^2+^-Binding Proteins and Cortical Development

### 3.1. Proliferation–Differentiation

During corticogenesis, NSCs/progenitor cells proliferate predominantly in the ventricular zone and subventricular zone, where RGCs and intermediate progenitors expand the progenitor pool and generate neuronal and glial lineages. These germinal zones provide the architectural and signaling milieu that supports cortical growth, regional patterning, and lamination [1].

**Figure 2:**
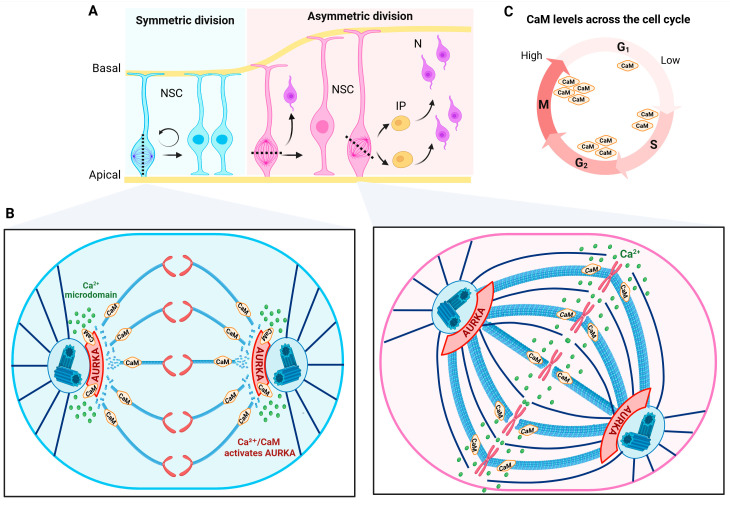
Calcium/calmodulin-dependent regulation in neural stem cells’ cell cycle. (**A**) Schematic representation of symmetric (left) and asymmetric (right) divisions of neural stem cells (NSCs) in the developing cortex, which promote renewal and neuron (N) differentiation directly or through intermediate progenitors (IP), respectively. Dotted line indicates the division plane according to the ventricular surface and arrows the resulting cell type (**B**) The symmetric (left) and asymmetric (right) division patterns are accompanied by remodeling of microtubule organization and spindle architecture in a Ca^2+^/calmodulin (CaM)-dependent manner by the activation of aurora kinase A (AURKA). Green dots represent specific Ca^2+^ domains that depend on the NSCs division pattern to determine microtubule density and stability (denser and more stable MTs in asymmetric proliferation). (**C**) Dynamic changes in calmodulin (CaM) levels across the cell cycle. CaM abundance increases progressively from G_1_ through S to G_2_/M, peaking during mitosis. Created in https://BioRender.com/aiiw8bj (accessed on 18 April 2006).

During neurogenesis, NSCs transition from symmetric proliferative divisions to asymmetric neurogenic divisions, which is a process that depends on mitotic spindle reorientation [61]. Symmetric divisions are commonly associated with a spindle oriented perpendicular to the ventricular surface, whereas asymmetric divisions often exhibit oblique or parallel orientations, resulting in differential inheritance of fate determinants [62]. This transition is accompanied by remodeling of microtubule morphology and spindle architecture (Figure 2) [62,63].

Across the cell cycle, the centrosome plays a central role in microtubule nucleation [64]. The centrosome duplicates during interphase, matures through recruiting γ-Tub ring complexes, and increases its microtubule-nucleation capacity [65,66]. Duplicated centrosomes then migrate to opposite poles and reorganize the interphase microtubule array into a bipolar mitotic spindle, enabling accurate chromosome alignment and segregation [67,68].

Microtubule architecture differs between the proliferative and differentiative NSC states. In early corticogenesis, abundant astral MTs support vertical divisions and proper positioning of daughter cells. During differentiation, spindle density increases and spindles more frequently adopt orientation and structural asymmetries associated with fate diversification (Figure 2A) [63,69].

Multiple studies suggest that rises in intracellular Ca^2+^ contribute to the functional regulation of the mitotic spindle. Classic work dating to the 1980s reported that elevated Ca^2+^ promotes microtubule depolymerization, leading to the hypothesis that Ca^2+^ remains relatively low during prophase to spindle assembly. At late metaphase and/or the onset of anaphase, a rise in Ca^2+^ concentration triggers the microtubule depolymerization necessary for chromosome segregation. In PtK-1 cells, the microinjection of Ca^2+^ (1–10 pM) during metaphase was reported to accelerate entry into anaphase, whereas EGTA microinjection, a Ca^2+^ chelator, blocked the metaphase-to-anaphase transition [70,71].

CaM abundance has been reported to increase from G1 through S to G2/M, with total protein levels rising across the cell cycle [72]. In mouse C127 cells, transient CaM elevation accelerated proliferation, whereas CaM reduction via antisense approaches induced transient cell cycle arrest, indicating that both G1 progression and mitosis are sensitive to CaM levels [8] and suggesting that CaM can be rate-limiting for cell-cycle progression under basal growth conditions and may be particularly influential at the G1-to-S transition (Figure 2B).

In HeLa cells, CaM localizes to the centrioles and the cytokinetic furrow, and its inhibition with W7 disrupts spindle organization [73,74]. CaM has also been reported to increase microtubule sensitivity to Ca^2+^-induced depolymerization, potentially via interactions with spindle-associated proteins, including a 52 kDa CaM-binding protein that colocalizes with CaM during mitosis in multiple cell lines [75]. The spindle disruption induced by W7 indicates that CaM activity is required for mitotic progression and supports the idea that, in the absence of Ca^2+^/CaM signaling, cells fail to transit from a disorganized microtubule to a functional bipolar spindle.

The Ca^2+^/CaM complex also regulates Aurora-A kinase (AURKA), which is implicated in both primary cilia disassembly and mitotic spindle functions. Disruption of the CaM–AURKA interaction compromises mitotic progression and cytokinesis, whereas mutation of CaM prevents its binding to AURKA, leading to defects in both processes (Figure 2C) [9].

Although many fundamental studies were performed in non-neural cell lines (e.g., PtK-1, HeLa), they established core principles regarding the Ca^2+^-dependence of microtubule dynamics and cell cycle transitions. Subsequent work in NSCs supports conservation of these mechanisms and highlights their relevance to corticogenesis.

It has been suggested that PCDH19 contributes to cell-cycle regulation during corticogenesis. PCDH19 is highly expressed in NSCs, and its silencing in vitro and in vivo induces aberrant spindle orientation and premature neurogenesis, consistent with a role in the temporal control of cell differentiation [47,76]. In zebrafish, loss of pcdh19 increases cell proliferation and neuronal production in the optic tectum [47]. Emond and colleagues (2021) performed a proteomic analysis to define the interactome of zebrafish pcdh19 following heterogenous expression in HEK293 cells. GO enrichments highlighted Cytoskeletal Protein Binding (Molecular Function), Microtubule Cytoskeleton (Cellular Component), and Establishment of Cell Polarity (Biological Process) as the most prominent categories. Within the “Microtubule Cytoskeleton” term, the authors identified Pcdh19-interacting proteins expressed in neural precursors and implicated in cell-division control. They further validated the interaction between Pcdh19 and neural precursor cell-expressed developmentally down-regulated protein 1 (NEDD1) in HEK293 cells, motivated by a priori evidence linking NEDD1 to proliferation, apoptosis, neural tube morphogenesis in zebrafish, and chromosomal microtubule nucleation and spindle function downstream of AURKA signaling in HeLa cells [77,78].

In the developing mouse brain, *Pcdh19 loss* does not produce gross morphological defects as in zebrafish; instead, an apparent increase in neuronal migration has been reported [48], suggesting partial functional compensation by other protocadherins. The role of PCDH19 in migration is discussed below.

Direct experimental evidence is still required to delineate the functional relationship between Ca^2+^ transients and PCHD19 in the proliferation and fate allocation of cortical NSCs. In particular, cortical lamination abnormalities and shifts in layer-specific neuronal phenotypes could reflect deregulation of symmetric versus asymmetric division, an aspect not fully resolved in *Pcdh19* knockout mice, in part because comprehensive layer–marker analyses were not performed. Moreover, systematic testing of additional candidate interactors identified by Emond and colleagues, especially proteins implicated in centrosome function, mitotic spindle assembly, and cytokinesis, such as CDK5RAP2, PLK1, PRC1, RACGAP1, and ARHGEF2, would help determine whether Ca^2+^ acts directly through PCDH19-dependent adhesion/signaling or indirectly via downstream pathways to regulate NSC proliferation and differentiation. Such analyses would also clarify whether Rho GTPase signaling, as proposed by this group, represents a key mechanism between PCDH19 and cytoskeletal control during corticogenesis [51].

Evidence also supports a role for CENs in NSCs/RGCs symmetry versus asymmetric division. CEN2 is required for centriole formation during S phase and for the formation of Ca^2+^-sensitive fibers linking mother and daughter centrioles. Changes in Ca^2+^ levels can trigger contraction or reorganization of these fibers, facilitating centrosome maturation before asymmetric division. Disruption of Ca^2+^-dependent CENs function impairs centriole duplication, promoting cell cycle arrest and/or genomic instability. Notably, while CEN2 is centrosome-associated in proliferating NSCs, its expression changes upon differentiation, with selective expression in subsets of mature astrocytes and broader cytoplasmic distribution. Hence, CEN2 has been reported in adult human brains, where it is expressed in a large fraction of astrocytes, but not in murine tissues, where it is largely restricted to ependymal cells, suggesting potential species differences and proposing CEN2 as a candidate astrocyte marker in human health and disease [79].

Furthermore, a study using a photoconvertible fluorescent protein fused to CEN1 reported that, in RGCs, mother centrosomes are preferentially inherited by self-renewing RGCs that remain in the VZ. In contrast, the daughter centrosomes are more frequently inherited by differentiating progeny that migrate away from the VZ toward the cortical plate [80].

Together, these findings suggest that centrosome inheritance is tightly regulated and coupled to fate specification during asymmetric division of NSCs in the developing neocortex. In humans, differential expression of CEN isoforms (e.g., CEN1 vs. CEN2) may further be associated with lineage allocation toward neuronal versus astroglial differentiation and maintenance.

### 3.2. Neuronal Migration

During corticogenesis, neuronal migration begins with the displacement of early-born neuroblasts from the VZ toward the pial surface along RGC scaffolds, which is a process regulated in part by reelin [81]. Migration entails coordinated morphological transitions driven by cytoskeletal remodeling. Neuroblasts initially adopt a multipolar morphology, extending and retracting multiple neurites until they become bipolar, with a leading process that engages the RGC fiber and a trailing process that accompanies soma translocation during radial migration. Upon reaching the appropriate cortical layer, neurons undergo terminal translocation, in which the soma advances, and the cell detaches from the RGC scaffold, thereby consolidating neuronal polarity and converting the leading process into an apical dendrite [82].

**Figure 3:**
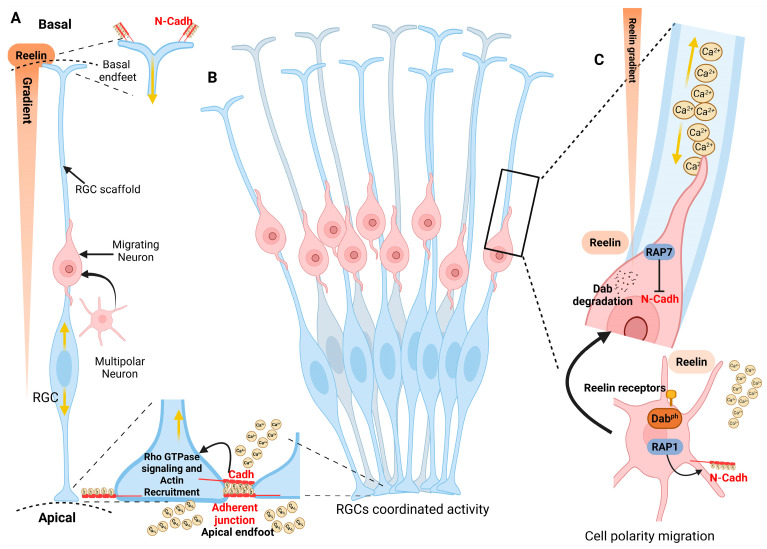
Ca^2+^-regulated radial glial coordination and neuron polarity during neuronal migration. Schematic representation of the coordinated activity of radial glial cells (RGCs) that guides neuronal migration during cortical development. RGCs extend long radial processes from the apical to the basal surface, forming a scaffold along which newborn neurons migrate. (**A**) At the apical and basal surfaces, extracellular signals, such as the reelin gradient, contribute to neural positioning. At the apical surface, RGCs are interconnected through Cadh-containing adherent junctions (N-Cadh/Pcdh19) that maintain epithelial integrity. Migrating neurons initially exhibit a multipolar morphology, then transition to a bipolar morphology and attach to RGC fibers to facilitate locomotion. (**B**) Groups of RGCs operate in coordinated clusters, forming functional microdomains consistent with the proposed “honeycomb” organization of the developing cortex. Within these radial units, intracellular Ca^2+^ signals propagate along radial glial processes, synchronizing their activity. (**C**) The reelin pathway through Rap1 and N-Cadh promotes multipolar neurons. Subsequently, its inactivation, due to degradation of phosphorylated Dab1 or inactivation of reelin receptors, decreases N-Cadh via the Rab7 pathway for bipolar morphology. Yellow arrows indicate the direction of the Ca^2+^ transient. Created in https://BioRender.com/aiiw8bj (accessed on 18 April 2026).

Although numerous intracellular CaBPs directly regulate the cytoskeleton during migration, this section emphasizes plasma membrane-associated Ca^2+^-dependent adhesion and signaling mechanisms that mediate neuroblasts-RGCs interactions. Ca^2+^ actively participates in neuronal migration during the early corticogenesis in mice (E10.5–E14.5). Using ex vivo anterior brain slices, the genetically encoded Ca^2+^ indicator GCaMP5G, and two-photon microscopy, Ca^2+^ transients have been reported to propagate bidirectionally along RGC fibers, suggesting Ca^2+^-mediated communication across the VZ, the intermediate zone, and the pial surface. Moreover, the amplitude and frequency of Ca^2+^ activity provide positional information that contributes to appropriate laminar allocation. In this framework, a “honeycomb” model has been proposed in which coordinated activity across RGCs assembles support for the columnar organization of the cerebral cortex (Figure 3) [10].

RGC polarity and scaffold integrity depend in part on cadherin-mediated adhesion that is intrinsically Ca^2+^-dependent. Binding extracellular Ca^2+^ to specific sites with cadherin ectodomains stabilizes an extended, rigid conformation required for homophilic adhesion between adjacent cells; in the absence of Ca^2+^, these regions collapse, compromising adhesive function. In COS cells, fluorescence resonance energy transfer measurements have shown that Ca^2+^ stabilizes N-cadherin (N-Cadh) interactions, thereby promoting robust intercellular adhesion. In contrast, lowering extracellular Ca^2+^ significantly weakens these interactions, underscoring the Ca^2^-dependent nature of N-Cadh-mediated adhesion (Figure 3) [83].

During neuronal differentiation, neuroblasts extend immature neurites, and suppressing N-Cadh impairs this outgrowth, indicating a requirement for early morphogenesis. Kawauchi et al. (2010) showed that Rab5-, Rab11-, and Rab7-dependent endocytic pathways regulate distinct phases of cortical migration through N-Cadh trafficking. Rab5 promotes N-Cadh delivery to neuronal processes; Rab11 mediates recycling; and Rab7 targets N-Cadh for lysosomal degradation, collectively influencing migration dynamics and dendritic maturation [84].

Reelin further promotes multipolar migration and the transition of multipolar to bipolar neuroblasts by activating Rap1 and increasing N-Cadh abundance at the cell surface, where N-Cadh acts in cis to stabilize FGFR and enhance Erk1/2 signaling, thereby supporting neuroblast migration [85]. N-Cadh and R-Cadh have also been implicated in nucleokinesis through interacting with β-catenin. Upon dephosphorylation by PTP1B, it forms a β-catenin complex with α-catenin and actin, enabling anchoring and transmission of contractile forces. This mechanism intersects with LIS1-dependent regulation, consistent with the integration of cadherin-based adhesion and microtubule-dependent machinery during nuclear translocation [86].

As previously mentioned, PCDH19 has been implicated in neuronal migration within the cortex. Because *PCDH19* is X-linked, random X-chromosome inactivation in heterozygous females generates cellular mosaicism that disrupts homophilic adhesion, alters network assembly, and contributes to cortical malformations and epilepsy phenotypes [46,87]. Additionally, *PCDH19* loss increases seizure susceptibility, linking developmental defects to clinical features observed in *PCDH19*-associated epilepsy [87,88]. Pcdh19 has emerged as a key regulator of neuronal migration during corticogenesis by coupling cell–cell adhesion to cytoskeletal dynamics. Loss-of-function studies indicate that Pcdh19 deficiency disrupts radial migration, leading to impaired cortical layering and abnormal neuronal positioning by modulating adhesion complexes that influence intracellular signaling pathways controlling cytoskeletal organization, likely through interactions with Rho GTPase signaling (Figure 3), thereby affecting actin remodeling and indirectly influencing microtubule stability and nucleokinesis. Additionally, altered Pcdh19 function has been associated with defects in centrosome positioning and polarity establishment, processes that are essential for coordinated neuronal movement. A proposed model is that Ca^2+^-dependent conformational changes in Pcdh19 regulate its adhesive properties and downstream signaling, linking extracellular cues to intracellular pathways that coordinate actin–microtubule crosstalk. However, while these findings support a role for Pcdh19 in migration, much of the mechanistic evidence remains indirect or derived from in vitro or non-cortical systems. Hence, further in vivo studies are required to establish how Pcdh19-dependent signaling specifically integrates Ca^2+^ dynamics with cytoskeletal remodeling during cortical neuron migration [45].

Recently, calneurons (CALNs) have been proposed to influence neuronal migration. These are transmembrane CaM-like neuronal Ca^2+^ sensors with high Ca^2+^ affinity, predominantly localized to the trans-Golgi network. CALN1 (CaBP8) and CALN2 (CaBP7) have been reported to exhibit among the highest Ca^2+^ binding affinities of neuronal sensors (Kds of 230 nM and 180 nM, respectively) [89,90,91]. In human-embryonic-stem-cell-derived models, *CALN1* has been linked to schizophrenia-associated loci. Its deletion in human dorsal forebrain organoids reduces mature neuronal output and Cajal–Retzius cell abundance, perturbs dorsal progenitor differentiation, and decreases *S100B* expression. These effects are likely mediated, at least in part, through downstream cytoskeletal pathways that converge on microtubule organization and neuronal polarity, and suggest that CALN1 may indirectly influence neuronal migration by shaping key cellular populations and signaling environments required for cortical organization [92].

### 3.3. Neurite Outgrowth and Early Neurite Maturation

Formation of neuronal circuits depends on neurite initiation, elongation, and guidance, which are processes executed by the growth cone. Growth cones are highly dynamic structures at the tips of developing axons and dendrites that integrate biochemical and mechanical cues to steer neurite extension toward synaptic targets. Within growth cones, Ca^2+^ acts as a spatiotemporally precise regulator, linking extracellular guidance signals to cytoskeletal remodeling by activating effector proteins that decode Ca^2+^ transients into defined structural outputs. Importantly, the amplitude, source, spatial distribution, and kinetics of Ca^2+^ transients are critical determinants of growth cone motility and directional decision-making. Although Ca^2+^-dependent actin remodeling and membrane trafficking are essential for growth cone motility, these processes are functionally coupled to microtubule invasion into the peripheral domain, which stabilizes directional growth and supports neurite extension.

**Figure 4:**
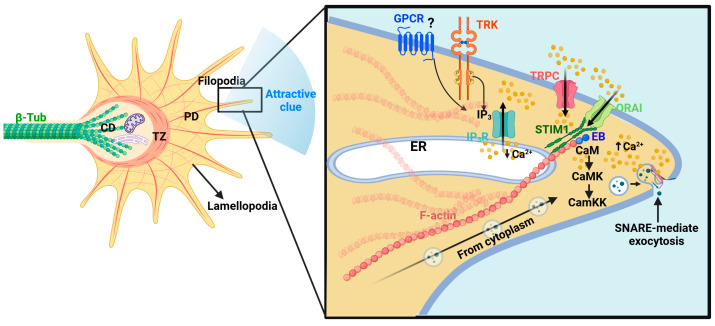
Ca^2+^ microdomain signaling in growth cone filopodia during guidance cue responses. Schematic of a neuronal growth cone highlighting the central domain (CD), transition zone (TZ), and peripheral domain (PD), with lamellipodia and filopodia at the leading edge. Inset: magnified single filopodium illustrating Ca^2+^ microdomains generated by activation of membrane receptors, including G-protein-coupled receptors (GPCRs) and tropomyosin receptor kinases (TRK). Receptor signaling promotes production of inositol 1,4,5-trisphosphate (IP_3_), triggering Ca^2+^ release from the endoplasmic reticulum (ER), and drives Ca^2+^ influx through stromal interaction molecule (STIM1) interactions with store-operated Ca^2+^ entry channels (ORAI) and transient receptor potential canonical (TRPC) channels. Local Ca^2+^ elevations activate Ca^2+^-dependent effectors, such as calmodulin (CaM) and Ca^2+^-calmodulin-dependent kinases (CaMK/CaMKK), as well as STIM1/microtubule plus-end-tracking protein (EB), leading to actin cytoskeleton remodeling that regulates filopodial protrusion and retraction, growth cone motility, and directional turning. At the filopodial tip, Ca^2+^-dependent vesicle trafficking and SNARE-mediated exocytosis promote asymmetric membrane addition to reinforce guidance cue-directed steering. Created in https://BioRender.com/aiiw8bj (accessed on 18 April 2026).

The growth cone is organized into a peripheral domain enriched in F-actin-based lamellipodia and filopodia, a central domain containing stable microtubule bundles and organelles such as the ER, and a transition zone that supports Ca^2+^-dependent molecular switches between advance, turning, and retraction. At the leading edge, localized Ca^2+^ transients can promote actin polymerization and membrane protrusion in response to attractive cues (Figure 4) [93,94,95]. In contrast, global and/or high-amplitude Ca^2+^ elevations activate actin severing and depolymerizing pathways, including gelsolin and the phosphatase CaN, which can regulate cofilin-mediated F-actin disassembly [96,97]. Ca^2+^ influx also modulates myosin II activity, and thus retrograde actin flow, which pulls the cytoskeleton rearward unless counterbalanced by substrate adhesion [98,99].

Within filopodia, localized increases in Ca^2+^ activate calpains, which are Ca^2+^-dependent proteases that remodel adhesion and tune the balance between kinase- and phosphatase-mediated signaling, thereby stabilizing protrusions. Calpain-mediated proteolysis of adhesion-associated proteins, including talin and focal adhesion kinase, contributes to the adhesion turnover required for guidance decisions [100].

Concomitantly, the invasion of dynamic MTs from the central into the peripheral domain stabilizes the newly selected direction of growth. This process is coupled to ER Ca^2+^ dynamics and to the interaction of Stim1 with microtubule plus-end-tracking proteins EB1 and EB3, which promotes coordinated recruitment of dynamic MTs and ER tubules toward the proximal side of filopodia exposed to attractive stimuli (Figure 4) [101]. Beyond Stim1, the microtubule-associated protein tau facilitates exploration of the actin-rich peripheral domain. Tau knockdown yields disorganized MTs that fail to invade filopodia and impair growth cone navigation [102]. Consistent with this model, *Stim1* mRNA silencing in embryonic rat dorsal root ganglion sensory neurons restricts ER tubule extension into filopodia, depletes local Ca^2+^ stores, and abolishes the directional growth cone turning. In zebrafish embryos, loss of Stim1a function in motor neurons reduced filopodia density, induced axonal stalling at choice points, and diminished Ca^2+^ transients during in vivo navigation [101].

A major route by which Ca^2+^ regulates growth cone decision-making is through phosphorylation-dependent signaling cascades. Wayman et al. (2004) demonstrated in neonatal hippocampal neurons and postnatal cerebellar granule neurons that Ca^2+^/CaM activates CaMKK and CaMKI, thereby regulating growth cone motility and axon extension [103]. More broadly, relatively higher Ca^2+^ levels can favor CaMKII activation and are frequently associated with attractive turning, whereas lower Ca^2+^ levels preferentially engage CaN-dependent pathways linked to repulsive responses [104]. Recent optogenetic approaches refine this framework by showing that Ca^2+^ frequency and amplitude function as separable coding parameters: slow, low-amplitude signals predominantly regulate steering direction, whereas fast, high-amplitude signals influence the rate of axon extension [105].

Thus, Ca^2+^ signaling integrates actin dynamics with microtubule remodeling, particularly microtubule invasion into the peripheral domain to ensure coordinated cytoskeletal organization during neurite guidance.

In addition to cytoskeletal remodeling, Ca^2+^-dependent membrane trafficking is required for neurite initiation and extension. The ER contributes to Ca^2+^-dependent regulation of vesicle-associated membrane proteins (VAMPs) and SNARE-mediated fusion events. VAMP2 and VAMP7 have been implicated in early neurite sprouting from the soma, whereas subsequent outgrowth is supported by VAMP7-positive vesicles transported from soma to growth cone in a microtubule-dependent manner (Figure 4) [106,107]. Additional SNARE components, including SNAP25 and syntaxins 1, 6, and 13, are also required for axon outgrowth [108,109,110,111].

Taken together, the aforementioned studies indicate that Ca^2+^ signaling does not simply activate general kinase pathways; rather, it spatially organizes cytoskeletal and trafficking regulators within compartmentalized microdomains to coordinate protrusion, adhesion turnover, and microtubule invasion.

Collectively, Ca^2+^ amplitude, source, localization, and temporal dynamics are integrated through CaMKII–CaN switching, calpain-mediated adhesion remodeling, SOCE-dependent signaling, ER–microtubule coupling, and Ca^2+^-regulated exocytosis to drive coordinated rearrangements of actin, MTs, and membrane. This integrated architecture enables growth cones to convert extracellular gradients into directional motility, ensuring accurate axon pathfinding during circuit formation.

**Figure 5:**
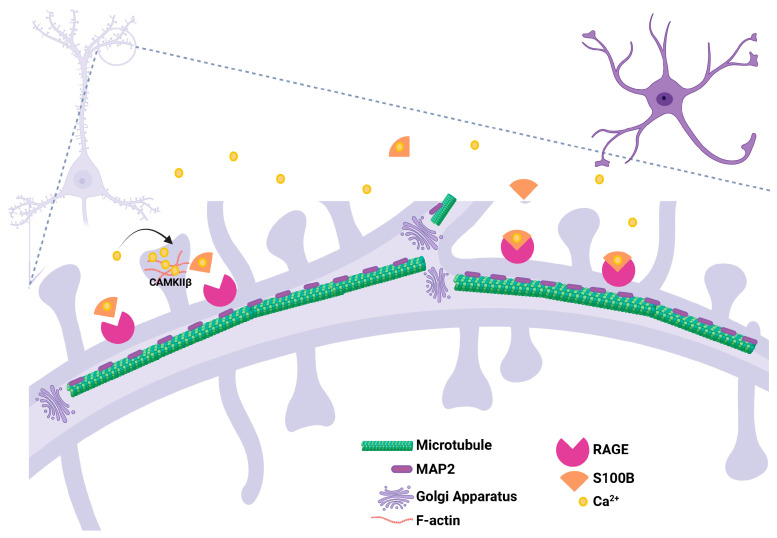
Calcium-dependent regulation of dendritic development and maintenance. During CNS development, astrocytes release S100B, a Ca^2+^-binding protein that acts as a paracrine modulator of neuronal morphogenesis. At physiological concentrations, S100B binds to RAGE (Receptor for Advanced Glycation End Products) expressed in developing neurons, activating intracellular signaling cascades including MAPK/ERK, PI3K/Akt, and NF-κB through Ca^2+^-dependent signal that control key processes in dendritic morphogenesis, including neurite extension, dendritic branching, and cytoskeletal remodeling. In this context, MAP2 (Microtubule-Associated Protein 2) plays a critical structural role in dendrites by stabilizing MTs and coordinating their interaction with actin filaments, which are processes that are highly sensitive to Ca^2+^ signaling. Ca^2+^ oscillations can modulate the activity of effector proteins such as calmodulin and Ca^2+^-dependent kinases, influencing cytoskeletal organization and the phosphorylation state of microtubule-associated proteins, including MAP2. The interaction among astrocyte-derived S100B, RAGE activation in neurons, and intracellular Ca^2+^ signaling regulates dendritic elongation, branching, and maturation, promoting dendritic arbor formation during the establishment of neuronal circuits in the developing brain. Created in https://BioRender.com/aiiw8bj (accessed on 18 April 2026).

Beyond growth cone steering and early axon pathfinding, Ca^2+^-regulated cytoskeletal programs continue to shape neurite maturation, including dendritic branching and spine stabilization, through coordinated regulation of actin-microtubule coupling and local microtubule dynamics. S100B has been implicated in cytoskeletal regulation during brain development through interaction with microtubule-associated proteins, including tau, thereby modulating microtubule stability and assembly. S100B may also influence neuronal structure via a CaMKII-dependent mechanism [35]. Double immunofluorescence studies report S100B colocalization with GFAP- and vimentin-positive cells, as well as association with centrosomes, supporting structural coupling to cytoskeletal elements. In transgenic mice overexpressing human S100B, increased MAP2-positive dendritic density has been observed during early hippocampal development, suggesting that S100B modulates dendritic architecture and may also affect synaptic maturation via microtubule reorganization (Figure 5) [112].

Consistent with a prominent role for CaMKIIβ in actin-based morphogenesis, shRNA-mediated silencing of *CamkIIβ* mRNA in CA1 pyramidal neurons in organotypic slices from postnatal rats (P6–P8) reduces mature dendritic spine density and increases immature filopodia-like protrusion. This phenotype is rescued by expression of an shRNA-resistant *CamkIIβ* variant, highlighting the requirement for CaMKIIβ/F-actin expression in dendritic spine maintenance [26]. CaMKII activation has been proposed to trigger a transient phase of robust actin remodeling following Ca^2+^ influx, followed by an autoinhibited phase that stabilizes induced structural changes. Notably, CaMKII has also been reported to associate with MTs near active synapses, supporting a dual role in coordinating actin and microtubule dynamics during synaptic plasticity [26,27].

In the rat brain, PCDH19 expression peaks around postnatal day 10 (P10), coinciding with dendritic maturation. In utero knockdown disrupts hippocampal pyramidal neuron migration and reduces dendritic length; these effects are rescued by shRNA-resistant PCDH19. The underlying mechanism has not yet been clearly defined, and although cytoskeletal regulation is proposed, its role remains unclear [48].

## 4. Conclusions

This review positions Ca^2+^ signaling as a central integrator of microtubule dynamics during corticogenesis, linking extracellular cues to the cytoskeletal mechanisms that govern neural progenitor behavior and cortical assembly. Through Ca^2+^-dependent effectors, including CaM/CaMK pathways, S100 proteins, cadherins/protocadherins, especially PCDH19, centrins, and ER Ca^2+^ sensors, spatiotemporal Ca^2+^ signals are coupled to centrosome function, mitotic spindle organization, and microtubule remodeling. Collectively, these processes regulate progenitor proliferation, division mode, neuronal migration, and early morphogenesis and morphological maturation of cortical neurons.

Despite these advances, a key unresolved question is how distinct Ca^2+^ signaling features, such as oscillations, frequency, local microdomains, and multicellular waves, are selectively decoded into specific microtubule-based outputs. Although several components of these pathways have been directly characterized in mammalian NSCs, many proposed mechanisms remain extrapolated from non-neural or simplified in vitro systems, underscoring the need for in vivo validation in the developing cortex. Addressing this gap will require integrative approaches that combine high-resolution Ca^2+^ imaging with quantitative measurements of microtubule dynamics and cell behavior in physiologically relevant models and preparations.

From a translational perspective, disruption of Ca^2+^-dependent cytoskeletal regulation is increasingly linked to neurodevelopmental disorders characterized by altered cortical cytoarchitecture and connectivity. A more precise understanding of Ca^2+^-microtubule signaling networks may therefore enable identification of tractable nodes for therapeutic modulation of neurogenesis and cortical organization. Overall, the evidence reviewed here supports a model in which Ca^2+^ signaling operates as a context-dependent regulator that integrates molecular signaling and cytoskeletal remodeling to shape corticogenesis.

## Data Availability

No new data were created.

## Abbreviations

The following abbreviations are used in this manuscript:

AURKA	Aurora-A kinase
Ca^2+^	calcium
CALN	calneuron
CaM	calmodulin
CaMKs	Ca^2+^/calmodulin-dependent kinases
CaN	calcineurin
CaBPs	calcium-binding proteins
CEN	centrin
CRAC	Ca^2+^-release-activated calcium channels
DAG	diacylglycerol
EC	extracellular cadherin domains
ER	endoplasmic reticulum
F-actin	filamentous actin
GPCRs	G-protein-coupled receptors
IPs	intermediate progenitors
IP3	inositol 1,4,5-trisphosphate
IP3Rs	inositol 1,4,5-trisphosphate receptors
MTs	microtubules
N-Cadh	N-cadherin
NECs	neuroepithelial cells
NSCs	neural stem cells
PCDH19	protocadherin 19
PLC	phospholipase c
RGC	radial glial cell
STIM	stromal interaction molecule
TRPC	transient receptor potential channels
VZ	ventricular zone
VAMPs	vesicle-associated membrane proteins
γ-Tub	γ-tubulin
